# A multiplex droplet digital PCR assay for chromosome copy-number determination in *Candida albicans*

**DOI:** 10.1093/g3journal/jkag201

**Published:** 2026-07-28

**Authors:** Eli Isael Maciel, Sylvain Ursuegui, Sana Ahmed-Seghir, Corinne Maufrais, Stéphanie Roy, Cécile Gautier, Philipp Brandt, Cécile Jovelet, Laras Pitayu, Iuliana V Ene

**Affiliations:** Fungal Heterogeneity Group, Department of Mycology, Institut Pasteur, Université Paris Cité, Inserm U1359, Paris 75015, France; Life Science Group, Bio-Rad Laboratories, Inc. (LSG PARIS), Villejuif 94280, France; Life Science Group, Bio-Rad Laboratories, Inc. (LSG PARIS), Villejuif 94280, France; Fungal Heterogeneity Group, Department of Mycology, Institut Pasteur, Université Paris Cité, Inserm U1359, Paris 75015, France; Bioinformatic Hub, Institut Pasteur, Université Paris Cité, Paris 75015, France; Fungal Biology and Pathogenicity Unit, Department of Mycology, Institut Pasteur, Université Paris Cité, Inserm U1359, Paris 75015, France; Life Science Group, Bio-Rad Laboratories, Inc. (LSG PARIS), Villejuif 94280, France; Fungal Heterogeneity Group, Department of Mycology, Institut Pasteur, Université Paris Cité, Inserm U1359, Paris 75015, France; Fungal Heterogeneity Group, Department of Mycology, Institut Pasteur, Université Paris Cité, Inserm U1359, Paris 75015, France; Fungal Biology and Pathogenicity Unit, Department of Mycology, Institut Pasteur, Université Paris Cité, Inserm U1359, Paris 75015, France; Life Science Group, Bio-Rad Laboratories, Inc. (LSG PARIS), Villejuif 94280, France; Life Science Group, Bio-Rad Laboratories, Inc. (LSG PARIS), Villejuif 94280, France; Fungal Heterogeneity Group, Department of Mycology, Institut Pasteur, Université Paris Cité, Inserm U1359, Paris 75015, France

**Keywords:** aneuploidy, copy-number variation, high-order multiplexing, color-combination encoding, Fungal2026

## Abstract

Chromosome copy-number variation is a major contributor to genome plasticity and adaptation in *Candida albicans*, a leading fungal pathogen of humans. Aneuploidy, defined as deviations from the normal diploid chromosome set, rapidly alters gene dosage and is often observed during exposure to host-imposed and antifungal stress. Accurate detection and quantification of chromosomal copy-number changes are thus essential to dissect the mechanisms by which *C. albicans* adapts and evolves. Here, we describe the development, optimization, and validation of a 6-color, 16-plex droplet digital PCR assay for simultaneous quantification of all *C. albicans* chromosome arms in a single reaction. Each target is detected by a unique dual-color or single-color combination of probes, enabling high-order multiplexing through binary fluorescence encoding. Following optimization of probe concentrations, PCR cycling parameters, genomic DNA extraction, and pretreatment with restriction enzymes, the assay provides accurate, reproducible chromosome-level copy-number estimates that correlate closely with whole-genome sequencing results across euploid and aneuploid isolates. Compared to whole-genome sequencing, the assay is rapid, cost-effective, and scalable, requiring minimal DNA input and allowing high-throughput analysis of large collections of isolates. The 16-plex assay thus provides a platform for dissecting genome instability and adaptive evolution in *C. albicans*.

## Teaser

This study developed and validated a 16-plex droplet digital PCR assay that estimates chromosome dosage across the entire genome of the human fungal pathogen *C. albicans* in a single reaction. The assay uses six fluorescent colors and unique color combinations to track one marker on each chromosome arm, enabling rapid detection of aneuploidy (extra or missing chromosomes). Results closely matched whole-genome sequencing for isolates with simple aneuploid forms and detected low-frequency trisomic clones in mixed populations. With optimized DNA preparation, this method provides a practical tool for screening for genome instability in research and clinical settings.

## Introduction

Aneuploidy, the presence of abnormal chromosome numbers, is a hallmark of genome instability and a major driver of adaptive evolution across biological systems (Torres et al. 2008; Li and Zhu 2022). In cancer, aneuploidy fuels tumor heterogeneity, promotes rapid adaptation to therapy, and contributes to disease progression (Vasudevan et al. 2021; Li and Zhu 2022). Karyotypic plasticity is particularly evident in human fungal pathogens, where aneuploidy is frequently associated with adaptation to host environments and antifungal stress (Selmecki et al. 2006; Rancati et al. 2008; Torres et al. 2008; Pavelka et al. 2010; Morrow and Fraser 2013; Ene et al. 2018; Stone et al. 2019; Kakade et al. 2023; Vande Zande et al. 2024; Wei et al. 2025; Heater et al. 2026; Lehmann et al. 2026). These examples illustrate that specific chromosome gains can confer measurable fitness advantages under environmental and drug-induced stress by altering gene dosage without requiring point mutations (Bennett et al. 2014; Tsai and Nelliat 2019; Todd and Selmecki 2020; Vande Zande et al. 2023).

Among fungal pathogens, *Candida albicans* exemplifies the dual nature of aneuploidy as both a source of genetic innovation and a contributor to phenotypic variability. *C. albicans* is an opportunistic commensal capable of causing life-threatening systemic infections in immunocompromised individuals (Brown et al. 2012; Pappas et al. 2018; Denning 2024). The genomic plasticity of this species underlies its ability to thrive in fluctuating host niches and in the presence of antifungal drugs (Selmecki et al. 2010). Unlike most fungi, *C. albicans* lacks a complete sexual cycle, limiting its ability to shuffle alleles through meiosis (Ene and Bennett 2014). Instead, it exploits single-point mutations, aneuploidy, ploidy shifts, loss of heterozygosity, and parasexual recombination to generate genetic diversity (Selmecki et al. 2010; Hickman et al. 2013; Morrow and Fraser 2013; Bennett et al. 2014; Forche 2014; Todd et al. 2019; Avramovska et al. 2022; Todd et al. 2023; Vande Zande et al. 2023).

Aneuploidy has been repeatedly observed in clinical isolates and experimental populations, often associated with adaptation to host niches and antifungal resistance. For example, monosomy of chromosome 5 (Chr5) promotes tolerance to fluconazole and caspofungin (Yang et al. 2019), trisomy of Chr2 confers cross-resistance to caspofungin, tunicamycin, and hydroxyurea (Yang et al. 2021a), while duplication of the left arm of Chr5 (iso-Chr5L) drives fluconazole resistance (Selmecki et al. 2006, 2008). Despite frequent fitness trade-offs associated with aneuploidy (Torres et al. 2007; Zhu et al. 2018; Tsai et al. 2019; Tsai and Nelliat 2019; Yang et al. 2021b), aneuploid isolates can also facilitate adaptation to mammalian host niches. Acquisition of a Chr7 trisomy enhances colonization of the murine gastrointestinal tract by increasing *NRG1* copy numbers and suppressing filamentation (Ene et al. 2018; Kakade et al. 2023). Concurrent trisomy of Chr5 and Chr6 increased fitness for the murine oral cavity and was associated with decreased epithelial adherence and invasion (Forche et al. 2018). These findings highlight aneuploidy as a powerful, reversible adaptive mechanism that enables *C. albicans* to fine-tune its physiology in response to environmental and host-imposed stresses, balancing the costs of genomic instability with context-dependent fitness gains that enhance survival, persistence, and pathogenic potential.

Accurate determination of chromosome copy number and identity is therefore critical for dissecting the role of aneuploidy in fungal adaptation. Whole-genome sequencing (WGS) remains the gold standard for this purpose, providing genome-wide resolution and quantitative accuracy. However, its cost, turnaround time, and computational requirements limit its use in large-scale or high-throughput studies. Alternative methods, such as flow cytometry and quantitative PCR (qPCR), provide faster and more accessible options but suffer from limitations. Flow cytometry detects total DNA content without chromosome identity, while qPCR is constrained by limited multiplex capacity and reliance on reference gene normalization. Early genome-wide aneuploidy studies in *Saccharomyces cerevisiae* used high-throughput qPCR to estimate chromosome copy number across multiple loci (Pavelka et al. 2010), but throughput in that approach was achieved by running many single-locus reactions in parallel rather than by multiplexing within a single assay. Moreover, qPCR quantification depends on normalization to a reference gene assumed to remain at constant copy number, an assumption often violated in aneuploid or structurally variable genomes. This dependence can introduce systematic bias when the reference region itself varies in dosage or amplification efficiency. In contrast, droplet digital PCR (ddPCR) quantifies absolute target concentrations by partitioning the reaction into thousands of droplets and applying Poisson statistics. Here, deviations are assessed relative to the genome-wide mean of all targets within the same sample, eliminating dependence on a predefined reference locus (Basu 2017; Majumdar et al. 2017). The fluorescence compensation and thresholding steps used here are fixed instrument calibrations rather than sample-specific adjustments and therefore remain constant across experiments.

ddPCR enables accurate quantification by partitioning a sample into thousands of nanodroplets in which amplification proceeds independently. Applying Poisson statistics to the fraction of positive droplets provides absolute copy-number estimates without the need for standard curves required in qPCR-based quantification. In conventional digital PCR (dPCR), each target is detected with a specific fluorescent probe in a single detection channel, so multiplexing capacity is constrained by the limited number of optical channels. To overcome this, higher-order multiplexing methods use different fluorescence intensities (amplitude- or ratio-based approaches) to encode multiple targets within the same channel. In these systems, 2 or more targets are distinguished by varying probe concentrations or fluorescence output, producing discrete intensity bands within that channel and allowing target quantification for the 2 nucleic acid fragments. However, these methods require complex multi-level thresholding and are less robust than color-combination multiplexing because fluorescence amplitude can vary between samples or runs, leading to threshold misplacement and imprecise quantification. An alternative approach is color combination, in which a unique color signature defines each target. While this is impractical in qPCR (because overlapping bulk fluorescence signals create ambiguity), dPCR can precisely identify color combinations in each partition and exclude ambiguous co-encapsulation events. This color-combination approach has been successfully implemented on 6-color and 7-color multiplex dPCR platforms, enabling detection of up to 15 and 21 targets, respectively. Importantly, the analysis remains simple and robust: only one threshold per fluorescence channel is required, and dedicated analysis software can automatically identify and quantify targets based on predefined color codes (Santos-Barriopedro et al. 2023). This approach substantially expands multiplexing capacity beyond conventional methods and minimizes competition between primer-probe sets.

In this study, we designed, optimized, and validated a 16-plex ddPCR assay for *C. albicans* that measures copy number across all 16 chromosome arms in a single reaction. The panel enables genome-wide quantification with single-reaction throughput and accuracy comparable to WGS. We describe the assay development process, including probe design and refinement of underperforming loci, optimization of DNA extraction and digestion, PCR cycling conditions, and calibration of fluorescence compensation. We then show that the assay detects mixed euploid-aneuploid populations and validate its quantitative precision by comparison with WGS-derived copy-number profiles across a diverse strain panel. Together, these results establish the 16-plex ddPCR assay as a rapid, scalable, and quantitative approach for genome-wide aneuploidy detection in *C. albicans*.

## Materials and methods

### *C. albicans* strains and growth

All experiments were performed using *C. albicans* isolates representing a range of karyotypes. The reference strain SC5314 (euploid A) served as one of the main euploid controls. For sensitivity testing in mixed populations, disomic and aneuploid subpopulations derived from the same genetic background (euploid B, CAY6440) were combined in defined ratios (25%, 50%, and 75% aneuploid). The collection of isolates used throughout the study is available in Supplementary Table 1. Yeast cells were cultured in YPD medium (1% yeast extract, 2% peptone, and 2% dextrose) overnight at 30 °C with shaking (200 rpm). Cells were collected from the stationary phase for genomic DNA extraction.

### Genomic DNA extraction

The yeast strains were grown on agar or liquid YPD medium for DNA extraction. Two extraction methods were evaluated for their effects on assay performance: a phenol–chloroform method and a Genomic-tip column-based kit (10223, Qiagen).

For phenol–chloroform extractions, 2 mL of an overnight culture or a patch of cells from a plate was washed twice with sterile water and resuspended in 400 µL TE (Tris-EDTA, pH 8) buffer containing 20 µL 20% SDS and 200 µL phenol:chloroform:isoamyl alcohol (25:24:1, neutral pH). Cells were lysed by bead beating (FastPrep, 3 cycles of 20 s at power setting 4, with a 3 min rest on ice between cycles). The aqueous phase was recovered, and the DNA was precipitated with ethanol and washed with 70% ethanol. After ethanol precipitation, the DNA pellet was resuspended in 800 µL of nuclease-free water and treated with 3 µL RNase A (10 mg/mL; R5503, Sigma) at 37 °C for 1 h. Following incubation, the DNA was re-precipitated with 1.5 mL 100% ethanol and 50 µL 3 M sodium acetate (pH 5.3).

Column-based extractions were performed according to the manufacturer's instructions (Qiagen). Briefly, 2 mL of an overnight culture or a patch of cells from a plate was washed with 1× TE buffer, the cell wall was digested with Lyticase (L2524, Sigma) in buffer Y1 for 1 h, followed by treatment with Proteinase K (P2308, Sigma) and RNase (R5503, Sigma) in buffer G1. The DNA was purified using Genomic-tip columns, eluted, precipitated with isopropanol, washed with 70% cold ethanol, and resuspended in sterile water.

### DNA digestion

Restriction enzyme digestion was tested using FastDigest enzymes (Thermo Fisher) BamHI (FD0054), EcoRI (FD0274), HindIII (FD0504), and PstI (FD0614). Each reaction contained 3 µg of genomic DNA, which was digested for 30 min according to the manufacturer's recommended conditions. Digested DNA was quantified and diluted to final template concentrations of 10 or 30 pg per reaction. Single-enzyme and combination digestions were tested systematically. SnapGene Viewer was used to verify enzyme restriction sites.

### Target selection and probe design

The *C. albicans* Crystal Digital PCR assay (R52002, Stilla Technologies, Bio-Rad Laboratories) was designed to quantify chromosome copy number at 16 loci, one per chromosome arm. Additional assay details, including recommended reaction concentrations, validated restriction enzymes, thermocycling programs, and instructions for image and data analysis, are provided in the assay information sheet (https://go.stillatechnologies.com/assay/c-albicans-crystal-digital-pcr-r-assay).

To minimize sequence or copy-number variation that might affect probe binding, candidate loci were chosen based on three principles: near diploid coverage in the SC5314 reference strain, proximity to centromeric regions, and the potential for high gene conservation due to essential or housekeeping function. Each target was verified against the SC5314 reference genome (assembly 22) to confirm the absence of restriction enzyme cut sites within the amplicon (Supplementary Table 2). The final targets, fluorophore combinations, and encoding schemes are listed in Supplementary Table 3. Probes used in this study were designed using Flex Probes and Universal Reporter Technology (Stilla Technologies, Bio-Rad Laboratories). Flex Probes are target-specific, non-fluorescent probe oligonucleotides that trigger the fluorescence of Universal Reporters in the presence of the targeted sequence. To design the oligonucleotides, the SC5314 assembly 22 was used. Sequence homology with species closely related to *C. albicans* (eg *Candida dubliniensis*, *Candida tropicalis*) was assessed using BLAST, and the primer and probe designs were adjusted accordingly. All primer/probe pairs were confirmed to be unique using BLASTn (E < 1e−3), allowing only a single perfect genomic match. Each target was first tested individually (simplex) and then as part of the 16-plex mixture. Simplex reactions yielded higher apparent copy numbers (reflecting reduced competition), but multiplex reactions preserved expected chromosome ratios and produced reproducible results after probe redesign and digestion.

### Reaction mix preparation and PCR cycling conditions

For each reaction, 0.6 µL Crystal Flex Probes and primers were mixed with 0.6 µL 10× Buffer A, 0.24 µL 10× Buffer B, 0.15 µL Universal Reporter A, 0.15 µL Universal Reporter B (all Naica PCR MIX reagents [R10106] and Universal Reporters 7 [R42401], Stilla Technologies, Bio-Rad Laboratories), and 4.3 µL of 14 or 42 pg/µL genomic DNA (gDNA) template. Primers and Flex Probes are listed in Supplementary Table 4. Five microliters of the prepared reaction mixture was loaded into each chamber of a Ruby Chip (C16011, Stilla Technologies, Bio-Rad Laboratories). The chip was loaded into the dPCR cycler (Naica Geode, Stilla Technologies, Bio-Rad Laboratories), a device that combines droplet generation and thermocycling. Following droplet partitioning, the amplification protocol was initiated, consisting of an initial denaturation at 95 °C for 3 min, followed by 60 cycles of 15 s at 95 °C for denaturation and 45 s at 60 °C for annealing and elongation. We empirically optimized the amplification program and found that 60 cycles yielded the best fluorescence separation between positive and negative droplet populations, especially at low DNA inputs (5 to 10 pg). In contrast, shorter programs (45 to 50 cycles) produced weaker signal intensities and less-defined cluster boundaries without improving specificity. The higher cycle number therefore enhances detection sensitivity and cluster resolution while maintaining assay specificity, as confirmed by no-template and simplex control reactions. A final elongation step at 58 °C for 15 min was performed before chip release. Alternative programs tested included extended elongation (60 s) and final elongation durations (10 or 15 min). Longer elongation at 60 s and a final elongation of 15 min improved separability scores, simplifying thresholding and improving left/right arm balance, especially for low-amplitude targets (eg Chr4R). Increasing probe concentrations from 0.5 to 1 µM further improved amplitude and reduced uncertainty.

To quantify the discrimination between positive and negative ddPCR droplets, we used a separability score (S), calculated by Crystal Miner that incorporates both the magnitude of signal separation and the variability within each droplet population (Supplementary Fig. 1). For each ddPCR reaction, droplets were partitioned into positive and negative populations based on fluorescence amplitude. The separability score was then calculated as


$$S=\frac{\mu_{\mathrm{pos}}-\mu_{\mathrm{neg}}}{\sqrt{\frac{\;p}{\;p-1}}\;\sigma_{\mathrm{pos}}+\sqrt{\frac{q}{q-1}}\;\sigma_{\mathrm{neg}}}$$

where $\mu_{\mathrm{pos}}$ and $\mu_{\mathrm{neg}}$ are the mean fluorescence amplitudes of the positive and negative droplet populations, respectively; $\sigma_{\mathrm{pos}}$ and $\sigma_{\mathrm{neg}}\;$ are the corresponding standard deviations; and *p* and *q* are the number of positive and negative droplets, respectively. A higher score indicates greater separation between positive and negative droplets, reflecting improved resolution. This metric provides a normalized measure of ddPCR performance that accounts for both signal dispersion and population size.

### Fluorescence detection and compensation

The chips were scanned using the dPCR reader (Naica Prism6, Stilla Technologies, Bio-Rad Laboratories), which is equipped with 6 fluorescence channels (blue, teal, green, yellow, red, and infrared [IR]). Exposure times were optimized for each channel: blue = 500 ms, teal = 800 ms, green = 250 ms, yellow = 350 ms, red = 1000 ms, and IR = 1,000 ms. Fluorescence compensation and spillover correction were implemented in Crystal Miner v4.0 software (Stilla Technologies, Bio-Rad Laboratories). Single-color control reactions were used to quantify bleed-through across channels, generating a spillover compensation matrix (Madic et al. 2016). In practice, users do not need to generate a new matrix manually for each experiment. The *C. albicans* Crystal Digital PCR assay includes a validated matrix pre-configured by the manufacturer and automatically applied by the software when a chip is read. The process is largely automated and intuitive: the software corrects spectral overlap in real time and displays compensated fluorescence plots for user inspection. A “run” refers to a complete chip processed within a single instrument session, and the same matrix can be reused across runs as long as the optical calibration of the reader remains unchanged. If desired, users can regenerate or edit the matrix by rerunning single-color controls. According to the manufacturer, each chip can be read up to three times without loss of fluorescence integrity. The validated matrix was applied consistently across all experiments. Compensation restored orthogonal cluster geometry and improved signal separation visible in 1D and 2D amplitude plots (Supplementary Fig. 3b and c). After fluorescence compensation, thresholds distinguishing positive and negative droplet populations were manually defined in Crystal Miner. For each target, the threshold was placed near the upper boundary of the negative droplet cluster, guided by the automatic suggestions of the software and visualized in one-dimensional amplitude plots (Supplementary Fig. 3b and c). This conservative placement minimizes false negatives while capturing true low-amplitude positives within the intermediate “rain” region, which typically reflects partial amplifications or co-encapsulated templates in high-multiplex ddPCR assays. This approach is consistent with current best practices for multiplex digital PCR analysis.

### Chromosome copy-number determination

For each ddPCR assay, the reaction mixture was partitioned into thousands of droplets, each functioning as an independent PCR reaction. Following amplification and fluorescence detection, droplets were classified as either positive or negative for each target. Assuming random partitioning of template molecules, the distribution of target occupancy across droplets follows a Poisson distribution. The absolute concentration of each target (copies/µL) was calculated using the Poisson equation: λ = −ln(1 − *p*), where *p* represents the fraction of positive droplets and λ the mean copies per partition. This correction accounts for multiple target molecules that may co-encapsulate within single droplets, minimizing systematic underestimation of copy number. This Poisson-based quantification is the standard analytical framework implemented in all major digital PCR software packages, independent of instrument manufacturer (Majumdar et al. 2017). Confidence intervals for each target concentration are derived from the binomial variance of the positive and negative droplet counts. Chromosome-arm copy numbers were then determined by normalizing each arm's absolute concentration to the genome-wide average signal across the 16 targets for each isolate. Examples of fractions of positive droplets, as well as replicate-level copy-number outputs, are available in Supplementary Fig. 5 and Supplementary Tables 5 to 7. More information regarding Poisson-based quantification in digital PCR can be found in Hindson et al. (2013), Majumdar et al. (2015, 2017), Whale et al. (2016), Basu (2017), Zhang and Noji (2017), and Santos-Barriopedro et al. (2023).

### WGS and coverage analysis

*C. albicans* cells were cultured in 5 mL YPD medium overnight at 30 °C with continuous shaking at 200 rpm. The DNA was extracted using the Qiagen Genomic DNA kit and Qiagen Genomic-tip20/G, according to the manufacturer's instructions. Sequencing libraries were prepared by SeqCenter (Pittsburgh, Pennsylvania) using the Illumina DNA Prep kit and sequenced on an Illumina NovaSeq 6000. The genome sequences and GFF annotation files for the SC5314 reference genome (haplotype A, A22-s07-m01-r130) were downloaded from the *Candida* Genome Database (Braun et al. 2005; Inglis et al. 2012) (http://www.candidagenome.org/). Reads were aligned to the reference genome using Minimap2 v2.17 (Li 2018). Sequence Alignment/Map tools (SAMtools) v1.10 (r783) (Li et al. 2009) and Picard tools v2.23.3 (http://broadinstitute.github.io/picard) were used to filter, sort, and convert the SAM files. Coverage levels were calculated using GATK DepthOfCoverage (McKenna et al. 2010). To estimate chromosome copy numbers, the read alignment depth was calculated for 1 kb windows across each genome and normalized to the average depth per genome. For published genomes, sequence information is included in Supplementary Table 1. Sequence read data for all other genomes have been deposited in the NCBI Sequence Read Archive under BioProject ID PRJNA1354400.

### Statistical analysis

For ddPCR quantifications, all isolates were analyzed in 3 to 5 independent biological replicates, each run separately; values shown represent means, with replicate results included in Supplementary Table 7. The correlation between ddPCR- and WGS-derived copy-number estimates was assessed using Pearson's correlation coefficient (*r*), with significance defined as *P* < 0.05.

## Results

### Design of a 16-plex chromosome-arm panel using 6-color combinations

To quantify the 8 *C. albicans* chromosomes, we developed a 16-plex ddPCR assay targeting one locus on each chromosome arm (Fig. 1a). The assay employs 6 fluorophores and assigns each chromosome arm/target a unique single- or dual-color code (Santos-Barriopedro et al. 2023) (Supplementary Table 3), allowing all 16 loci to be resolved within a single reaction using simple per-channel thresholding. Detection relies on Flex Probe chemistry: target-specific Flex Probes are non-fluorescent oligonucleotides that, upon target amplification, release a universal tag (Tag oligo) which hybridizes to a fluorophore-labeled Universal Reporter and is extended, generating the fluorescence signal used for droplet calling (Fig. 1b).

**Fig. 1.**
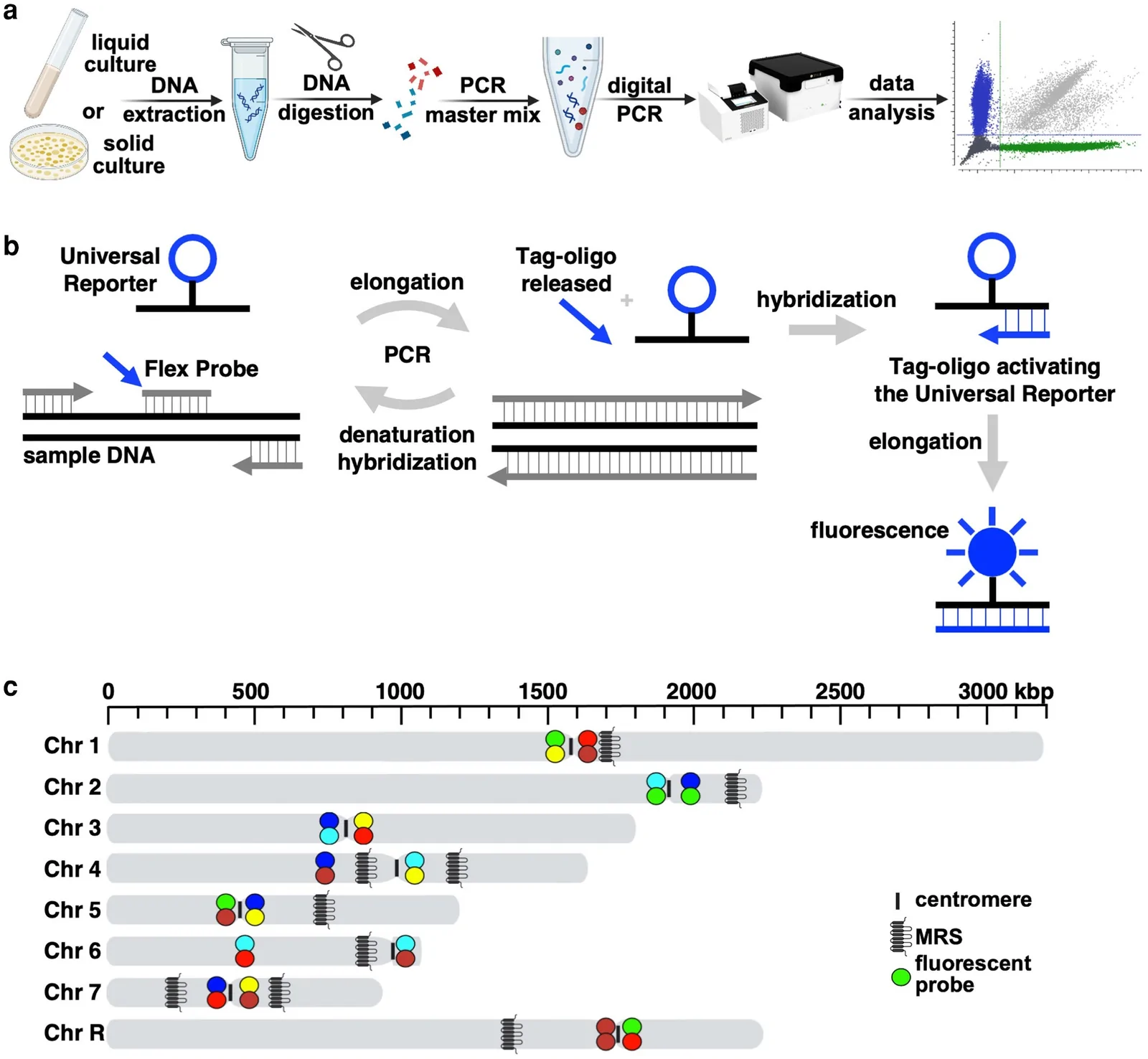
Assay design and fluorescence encoding strategy for 16-plex ddPCR chromosome copy-number profiling in *C. albicans*. a) Overview of the experimental workflow, including overnight growth of cells (liquid or solid medium), genomic DNA extraction, restriction enzyme digestion, reaction mix preparation, droplet digital PCR, and downstream data analysis. b) Flex Probe/Universal Reporter detection chemistry underlying the multiplexing strategy. Forward and reverse primers (gray arrows), Flex Probes (gray lines), universal reporters, and genomic DNA are combined in a single reaction. During amplification, target-specific Flex Probes are hydrolyzed to release a universal tag (Tag oligo) that hybridizes to a fluorophore-labeled Universal Reporter. Subsequent elongation generates the target-specific fluorescence signal used for droplet calling, enabling simultaneous discrimination of all 16 loci. c) Schematic of the 8 *C. albicans* chromosomes showing the positions of the 16 targeted loci (1 per chromosome arm). Centromeres and major repeat sequence (MRS) regions are indicated. Each arm is assigned a unique single- or dual-color code across 6 fluorescence channels to enable multiplex detection within a single reaction.

Candidate loci were selected based on normalized WGS coverage consistent with diploid levels at the target site, proximity to the centromere (to avoid telomeric regions prone to mutation and loss of heterozygosity), and association with essential or housekeeping functions (to reduce the likelihood of sequence divergence or structural variation that could impair probe binding). The resulting 16 targets (Fig. 1c) provide balanced chromosomal coverage and, because they were chosen from conserved regions, are expected to support consistent probe binding across genetically diverse isolates. As an initial performance check independent of strain background, we tested the panel using equimolar synthetic double-stranded DNA controls (gBlocks) for each chromosome arm. Under these controlled conditions, the measured arm ratios were close to the expected value of 1, supporting the accuracy of the target set and color-coding scheme. Together, this design establishes a scalable, single-reaction framework for genome-wide chromosome copy-number measurement in *C. albicans*.

### Probe redesign and PCR optimization improve multiplex cluster separation

Probes were initially designed as non-competing dual Flex Probe pairs, in which 2 differently labeled probes bind adjacent sites within the same amplicon (Santos-Barriopedro et al. 2023). Each target was first evaluated individually (simplex) and then combined into the full multiplex panel for single-reaction detection of all 16 loci. When tested on genomic DNA, simplex analysis of 2 strains (SC5314 [euploid A] and CAY8836-SC [Chr 1,4,5,7 3X]) revealed reproducible deviations from the expected R:L ratio for a subset of chromosomes: Chr1, Chr4, and Chr6 in SC5314 and Chr4 and Chr6 in CAY8836-SC (Supplementary Fig. 2a). These deviations persisted in multiplex runs, and an additional imbalance was observed for ChrR in both strains (Supplementary Fig. 2a). Inspection of 2D amplitude (dot) plots from simplex reactions localized these effects to probe performance. For SC5314, only 1 of the 2 probes designed for each arm produced efficient binding for Chr1, Chr4, and Chr6, whereas in CAY8836-SC, this behavior was restricted to Chr4 and Chr6 (Supplementary Fig. 2b).

Because each arm should contribute ∼50% of the double-positive signal, inefficient detection of one arm depletes the double-positive cluster and inflates single-color droplets, yielding an apparent arm imbalance. Notably, ChrRL is the only target encoded in a single channel (IR), and Chr6R generated a marked excess of IR-only droplets (Supplementary Table 5). Thus, a fraction of Chr6R molecules that should have contributed to the double-positive population were instead detected only in IR, biasing the inferred ChrR R:L ratio. We hypothesized that single-nucleotide polymorphisms (SNPs) and/or local DNA conformation impaired probe hybridization. To correct these probe-specific biases, we redesigned the probes for Chr1L, Chr4R, and Chr6R using a competing dual Flex Probe configuration, in which both probes target the same region and compete for hybridization (Santos-Barriopedro et al. 2023). For each target, the redesigned pair was based on the better-performing sequence from the original non-competing design.

Additional optimization steps included increasing the probe concentration from 0.5 to 1 µM and extending the final elongation step from 10 to 15 min. These adjustments increased signal amplitude and reduced stochastic detection variability, particularly under multiplex conditions. Consistent with this, average separability scores (indicating how well the positive and negative distributions are separated, Supplementary Fig. 1) improved after probe redesign and reaction optimization, yielding well-resolved positive and negative droplet populations across all targets. The longer final elongation improved separability (Supplementary Fig. 3a), likely by allowing additional time for the Tag oligo hybridized to the Universal Reporter to elongate and release the fluorophore. Because the optimal elongation time is assay-dependent, it can be adjusted to improve amplification efficacy and maximize cluster height, although overly long elongation steps may occasionally produce low-level positive droplets in the no-template control.

Together, these refinements produced distinct, non-overlapping signal clusters for all 16 loci within a single ddPCR reaction. Partition clustering analysis reliably separated positive and negative droplets, enabling direct absolute quantification of each target and robust genome-wide aneuploidy measurement in *C. albicans*.

### Spillover compensation improves multiplex droplet calling

Droplet calling and quantification were performed in Crystal Miner. Because the Naica system detects 6 fluorophores across 6 channels, partial spectral overlap (“spillover”) can distort droplet amplitudes and bias the multiplex quantification. Although each fluorophore is primarily excited and detected in its own channel, low-level excitation by other light sources and broad emission spectra can introduce measurable signal into non-primary channels. To correct this, we generated an assay-specific spillover compensation matrix using chambers containing single-color probe sets. For each fluorophore, we quantified the fraction of its signal detected in the other channels and used these values to build a spillover compensation matrix. The matrix was then inverted and applied to the raw fluorescence measurements to obtain spillover-corrected fluorophore intensities and to adjust for background fluorescence. This correction yielded cleaner one-dimensional amplitude distributions for thresholding (Supplementary Fig. 3b) and restored the orthogonal cluster geometry in 2D amplitude plots, improving separation between negative and positive droplet populations (Supplementary Fig. 3c). The resulting matrix can be saved and reused for all runs performed with this assay. After compensation, thresholds separating positive and negative droplets were manually set in each channel, and the population editor was used to define single- and dual-color codes for positive droplets of each target (eg *CCT2* in the blue and green channels; *SSK2* in the teal and yellow channels, Supplementary Fig. 3c). Using Poisson statistics, the software then estimated target concentrations and reported confidence intervals. Together, spillover correction and consistent droplet calling across runs improve high-order multiplex quantification.

### Column-based DNA extraction and higher DNA input improve 16-plex copy-number accuracy

To assess how genomic DNA preparation affects copy-number calling, we compared a widely used phenol–chloroform protocol with a commercial column-based kit (Qiagen) across 5 *C. albicans* isolates spanning euploid (SC5314), simple aneuploid (Chr3 1X, ChrR 3X), and complex aneuploid (Chr3,6 3X, Chr6,R 4X) karyotypes. Baseline chromosome copy numbers were first established from Illumina WGS (Fig. 2a). When these samples were analyzed with the 16-plex ddPCR assay, phenol–chloroform DNA produced noisier copy-number profiles and frequent deviations from the WGS karyotype (Fig. 2b). A likely explanation is that phenol–chloroform extraction can leave residual organic contaminants or partially oxidized phenolic compounds that inhibit droplet formation and/or polymerase activity (Nacheva et al. 2017). Electrophoretic comparison of DNA obtained by the 2 methods showed that phenol–chloroform preparations were more fragmented and exhibited artificially inflated spectrophotometric concentrations (Supplementary Fig. 4), leading to inconsistent template input and greater signal variability. In contrast, column-purified DNA yielded chromosome copy-number estimates that closely matched WGS across all isolates (Fig. 2b), reduced technical variance (from a mean SD of 0.71 to 0.38), and increased the fraction of positive droplets per reaction (from 3.25% to 21.21%) (Supplementary Fig. 5).

**Fig. 2.**
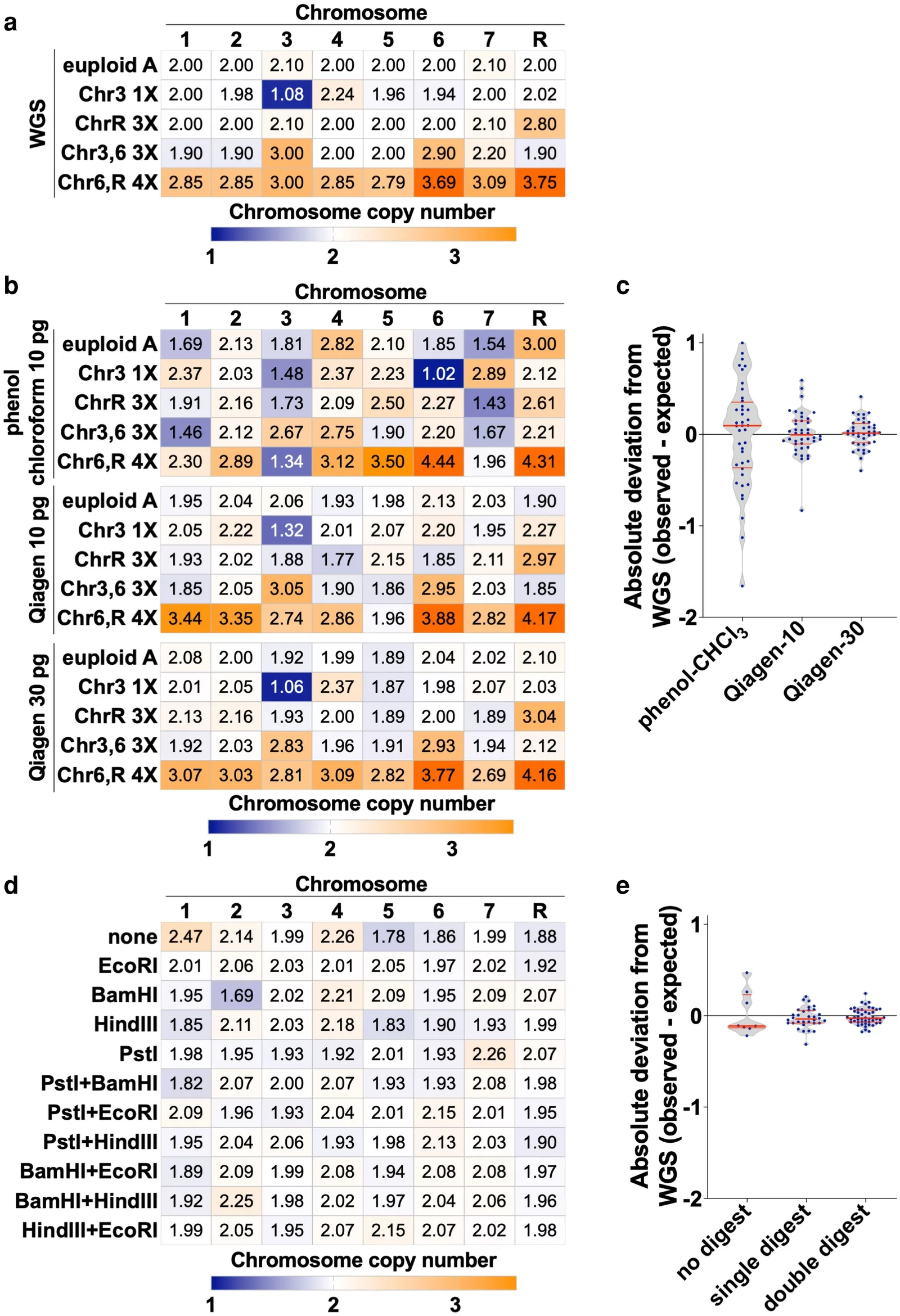
Genomic DNA preparation and restriction digestion improve 16-plex ddPCR accuracy. a) WGS-derived chromosome copy-number profiles for a reference euploid isolate and 4 aneuploid isolates representing simple and complex karyotypes. Values are shown as chromosome-level copy numbers. b) Heatmaps of ddPCR-derived chromosome copy-number estimates for the same isolates under different DNA extraction and input conditions, comparing phenol–chloroform extraction (10 pg/µL input) to column-based purification (Qiagen; at 10 pg/µL and 30 pg/µL). c) Violin plot showing absolute deviations from expected WGS-based copy-number values (observed − expected) under each extraction condition, demonstrating improved accuracy with column-purified DNA. d) Effect of restriction enzyme digestion on ddPCR accuracy using the euploid reference strain SC5314 (euploid A). Heatmaps show chromosome copy-number estimates obtained with no digestion, single-enzyme digests, or enzyme combinations. e) Violin plot showing absolute deviations from expected WGS copy numbers for undigested, single-, and double-digest conditions, illustrating that restriction digestion improves quantitative precision. For ddPCR quantifications, values correspond to averages across 3 biological replicates.

We next asked whether increasing DNA input could further improve assay precision. While 10 pg/µL of column-purified DNA produced acceptable profiles, raising the input to 30 pg/µL increased concordance with WGS (Fig. 2b and c), improved reproducibility across replicates (from a mean SD of 0.38 to 0.16), and further increased the positive droplet fraction from 21.21% to 38.7% (Supplementary Fig. 5). Based on these results, the optimized workflow uses 30 pg of column-purified gDNA per 5 µL reaction to maximize target detection and minimize false aneuploid calls.

### DNA digestion increases droplet resolution and copy-number accuracy in the 16-plex assay

High-molecular-weight genomic DNA can interfere with droplet formation and reduce amplification uniformity across targets. To test whether template fragmentation enhances assay performance, genomic DNA from the euploid reference strain SC5314 (euploid A) was digested with EcoRI, BamHI, HindIII, or PstI, either individually or in pairwise combinations, before amplification. Enzymes were selected to avoid predicted cut sites within the 16 amplicons (Supplementary Table 2).

Across conditions, digestion improved the accuracy of chromosome copy-number estimates relative to undigested DNA (Fig. 2d and e). While single-enzyme digests provided modest gains, enzyme combinations produced the most consistent improvements, particularly when PstI was combined with either EcoRI or HindIII. This was reflected by reduced variability between replicates in copy-number estimates and higher separability scores across chromosome arms (Supplementary Fig. 6a and b). Digestion also increased the fraction of positive droplets (Supplementary Fig. 6c), consistent with improved accessibility of amplifiable templates and reduced stochastic dropout. Undigested DNA tended to overestimate copy number for specific chromosomes, particularly for Chr1 and Chr4, whereas digestion corrected these biases, returning copy numbers closer to the expected disomic baseline (Fig. 2d and e; Supplementary Fig. 6d). Taken together, restriction digestion improved both droplet cluster resolution and quantitative accuracy. Consequently, we incorporated DNA pre-digestion with PstI + HindIII as a standard step in the optimized 16-plex workflow.

### The 16-plex assay sensitively detects mosaic aneuploidy and matches WGS across diverse karyotypes

To test sensitivity for detecting emerging aneuploid subpopulations, we generated defined mixtures of disomic and trisomic cultures from the same strain background (ChrR 2X:3X) at 25%, 50%, and 75% trisomic fractions. The assay revealed a progressive increase in ChrR copy number that closely matched the expected mixture averages, and reliably distinguished trisomy when only 25% of the population carried the extra chromosome (Fig. 3a). The same trend was observed at the chromosome arm level for RL and RR (Supplementary Table 6), indicating that the 16-plex format can resolve mosaic aneuploidy at low abundance despite the sampling noise inherent to droplet partitioning.

**Fig. 3.**
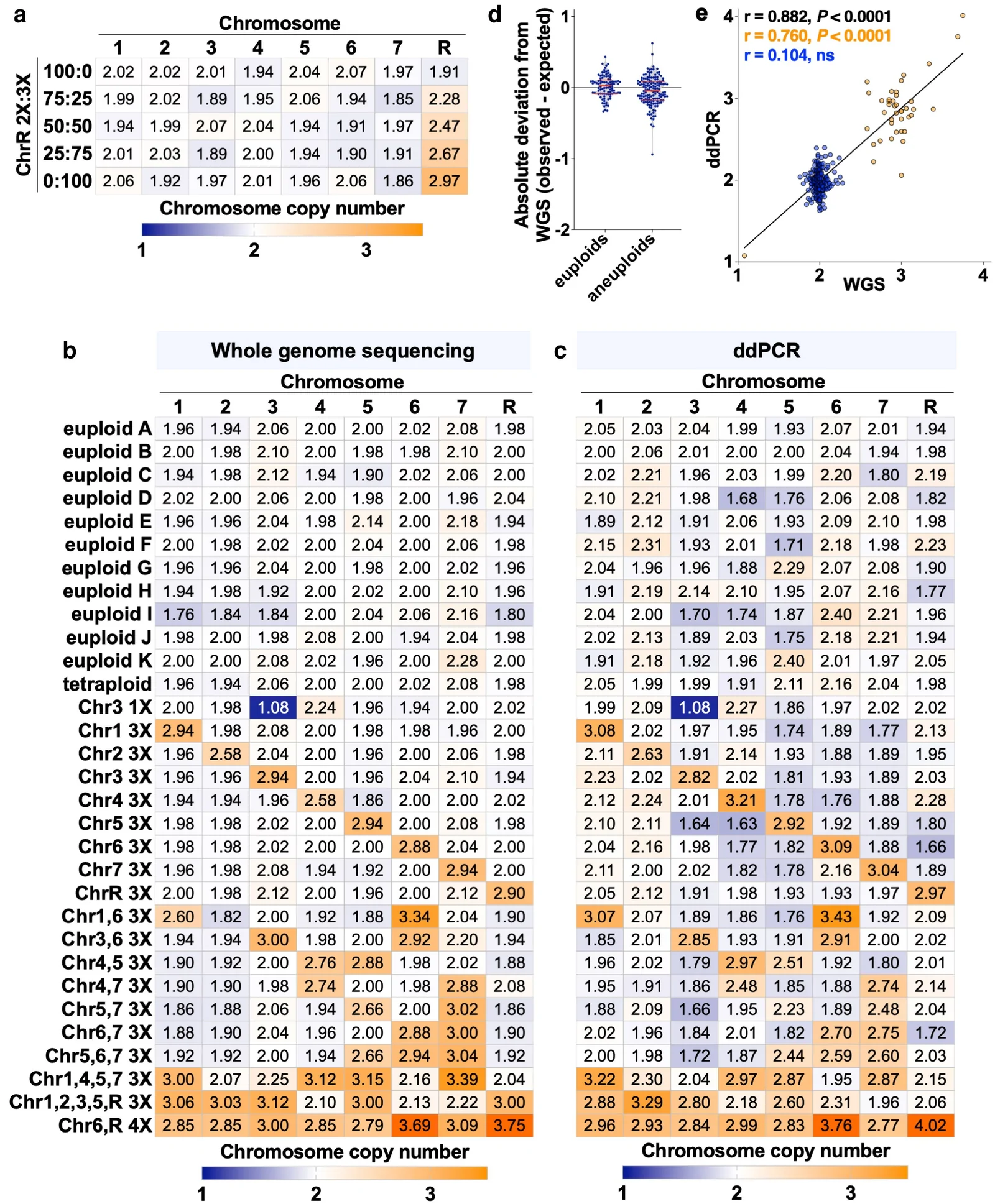
Assay performance across mixed populations and diverse isolate karyotypes. a) Detection of mosaic aneuploidy using defined mixtures of disomic (euploid B, ChrR 2X) and trisomic (ChrR 3X) subpopulations from the same background (euploid B, CAY6440) at defined ratios, showing progressive increases in average copy number. b) WGS-based chromosome copy-number profiles for 31 isolates, including 11 euploid, 1 tetraploid, and 19 aneuploid isolates from multiple genetic backgrounds. c) Corresponding ddPCR chromosome copy-number estimates for the same isolates, demonstrating strong quantitative concordance with WGS. d) Violin plot of absolute deviations between ddPCR- and WGS-derived values for euploid and aneuploid isolates. e) Correlation between WGS- and ddPCR-derived chromosome copy-number estimates. ddPCR measurements strongly agree with WGS across all chromosomes tested (Pearson's *r* = 0.882, *P* < 0.0001) and across aneuploid chromosomes specifically (orange; *r* = 0.760, *P* < 0.0001). Euploid chromosomes cluster near copy number 2 and show no significant within-group correlation because of their restricted dynamic range (blue; *r* = 0.104; ns, not significant). For ddPCR quantifications, values correspond to averages across 3 to 5 biological replicates.

We next assessed species specificity using genomic DNA from *C. dubliniensis* and *C. tropicalis*. Under standard cycling conditions, none of the 16 targets generated positive droplets, supporting assay specificity for *C. albicans* and suggesting that detection of related species would require redesigned primers/probes. To further evaluate the genetic stability of the probe-binding regions, we performed a sequence-diversity screen across 565 *C. albicans* isolates representing all major clades from multiple published datasets (Ford et al. 2015; Ropars et al. 2018; Rosenberg et al. 2018; Schutz et al. 2025). Across the 60 unique target regions, 36 (60%) were completely conserved, while the remaining 24 (40%) contained only 1 to 3 single-nucleotide polymorphisms, most of which were heterozygous and present at low frequencies (average minor-allele frequency ∼11%; Supplementary Table 9). Given the short length (∼20 bp) of the probes and the limited frequency of these variants, such polymorphisms are unlikely to impair hybridization or quantification efficiency. These results indicate that the majority of assay targets are genetically stable across the global *C. albicans* population, supporting robust performance across diverse genetic backgrounds.

Finally, we benchmarked ddPCR copy-number estimates against Illumina WGS across a panel spanning 11 genetically diverse euploid isolates, 1 tetraploid SC5314 derivative, and increasingly complex aneuploid karyotypes (Fig. 3b). Euploid and tetraploid isolates clustered tightly around the expected disomic baseline, and the assay correctly identified single-chromosome aneuploidies across all 8 chromosomes, including Chr3 monosomy and distinct trisomies (Fig. 3c). Isolates carrying 2 supernumerary chromosomes were also called accurately, with strong agreement between ddPCR and WGS (Fig. 3b to e) and consistent left/right arm estimates (Supplementary Table 7). In highly aneuploid isolates (4 or more altered chromosomes), ddPCR still captured broad copy-number elevations and flagged complex karyotypes, but complete resolution became less consistent, occasionally underestimating one affected chromosome, likely because calling becomes more challenging when many targets shift at once in highly aneuploid genomes (Fig. 3c and d).

Overall, these results show that the 16-plex ddPCR assay provides WGS-concordant copy-number calls for euploid, single-, and dual-aneuploid karyotypes, while remaining sensitive enough to detect low-frequency trisomic subclones and to flag isolates with more complex chromosomal alterations.

## Discussion

Here, we developed and validated a 16-plex ddPCR assay for genome-wide chromosome copy-number measurement in *C. albicans.* The assay combines digital quantification with high-order multiplexing through color-combination encoding across 6 fluorescence channels, enabling simultaneous readout of all 16 chromosome arms in a single reaction. Targeting both chromosome arms gives a built-in cross-check (left vs right) and helps capture large arm-scale copy-number changes that single-locus qPCR assays can miss. In practice, this workflow provides a rapid alternative to sequencing: from gDNA to copy-number calls can be completed within a day, without library preparation or bioinformatics.

As with any targeted assay, performance depends on the complementarity between probe and template. In genetically divergent isolates, locus-specific polymorphisms or small indels in primer/probe binding sites can reduce hybridization efficiency and bias local quantification. In addition, the resolution is limited to the assayed loci: partial aneuploidies that do not include the selected target regions will not be detected, and breakpoints cannot be mapped. Finally, high-order multiplexing requires stable fluorescence calibration. Spectral overlap and variability between replicates can affect cluster positions if compensation and thresholding are not standardized. These limitations can be mitigated by (i) selecting conserved targets (as done here) and redesigning individual probe sets when strain-specific dropout is observed, (ii) including a reference strain with a known karyotype, and (iii) using fixed instrument templates and a fixed spillover compensation matrix to stabilize cluster geometry across experiments.

Our screening of 565 genetically diverse *C. albicans* isolates revealed that most probe-binding regions are conserved across major clades and sources, including both clinical and commensal isolates. Only a minority of loci carried low-frequency heterozygous SNPs within binding regions, differences that are unlikely to substantially affect hybridization or quantification efficiency. Because the ddPCR assay is modular by design, any probe affected by lineage-specific polymorphisms can be readily redesigned and substituted while preserving the 16-plex configuration. This flexibility allows extension of the platform to new genomic targets, strain types, or even related *Candida* species, ensuring that the method remains robust and informative across genetically diverse isolates. Across a diverse isolate panel, the 16-plex assay showed strong agreement with WGS for euploid strains and for isolates carrying 1 to 3 supernumerary chromosomes, and it sensitively detected mosaic trisomy at 25% abundance in mixed populations. Performance was less consistent in highly aneuploid genomes, where many chromosomes deviate simultaneously, and accurate calling becomes more challenging. Even in such cases, the assay reliably flagged complex karyotypes and captured broad patterns of copy-number variation. Together, these results define a practical operating range: highly accurate for simple aneuploid states and informative for more complex karyotypes where follow-up by WGS may be warranted.

Relative to WGS, ddPCR offers advantages in speed, cost, and sample requirements. It is best suited for large-scale screening experiments where many isolates must be profiled quickly, as well as for settings where sequencing infrastructure is limited. A detailed cost and workflow comparison among ddPCR, qPCR, and WGS is provided in Supplementary Table 8, summarizing reagent and consumable costs, instrument access, processing time, and analysis requirements. This comparison highlights that ddPCR achieves genome-wide copy-number resolution at a fraction of the cost and turnaround time of WGS, while requiring substantially less DNA input and no downstream bioinformatics.

The assay's reliance on droplet partitioning also makes it compatible with low DNA inputs and heterogeneous populations. Although DNA quantity is not usually limiting for WGS of microbial cultures, the picogram-level input requirement may be useful when starting material is scarce, when isolates grow poorly, when only small colonies or low-yield clinically-derived material are available, or when many replicate or longitudinal samples must be screened rapidly. In these contexts, ddPCR is not intended to replace sufficiently high-coverage WGS, but rather to provide a rapid first-pass readout of relative chromosome dosage that can guide sample selection. WGS remains the preferred approach when base-pair resolution, unbiased genome-wide detection, or structural variant mapping is required. Overall ploidy (for example, haploid, diploid, or tetraploid) does not influence ddPCR performance because each locus is quantified independently and normalized to the genome-wide mean. Balanced haploid or tetraploid genomes therefore appear euploid by this method, while deviations reflect relative chromosomal dosage within a sample (a tetraploid example is presented in Fig. 3b and c). Absolute ploidy is therefore best assessed by orthogonal DNA-content measurements, such as flow cytometry. WGS can provide complementary evidence in some cases, but balanced whole-genome ploidy shifts may remain invisible to standard coverage-normalized analyses unless allele-frequency patterns, coverage calibrated against an appropriate reference, or other genomic features allow ploidy differences to be inferred. However, our optimization results underscore that standardized sample preparation is essential: column-based extraction, higher DNA input, and restriction digestion substantially improved positive droplet fraction, cluster separation, and copy-number accuracy.

A key strength of the assay is its modularity. Because the multiplex structure is encoded by probe sequences and fluorophore combinations, panels can be reconfigured to address new biological questions without altering the workflow. For example, the same design principles could be extended to track recurrent aneuploidies linked to drug resistance or adaptation, to include loci reporting focal amplifications, or to incorporate species- or clade-informative markers alongside copy-number targets. More broadly, by redesigning primer-probe sequences while preserving the color-combination logic, this approach should translate to other fungal species where copy-number variation contributes to stress resistance and host adaptation.

Overall, this 16-plex ddPCR assay bridges a practical gap between comprehensive but resource-intensive sequencing and low-plex assays. It provides an accessible platform for quantitative monitoring of genome instability, enabling rapid, scalable readout of karyotypic variation in both experimental and clinical contexts.

## Supplementary Material

jkag201_Supplementary_Data

## Data Availability

Sequence read data generated in this study have been deposited in the NCBI Sequence Read Archive under BioProject PRJNA1354400. Accession information for previously published genomes analyzed here is provided in Supplementary Table 1. Strains are available upon request. All data necessary to support the conclusions are present within the article, figures, and tables. Supplemental material available at G3 online.
