# Supplementary material for: A multiplex droplet digital PCR assay for chromosome copy-number determination in Candida albicans
Source: G3 (Bethesda). 2026 Jul 28;16(10):jkag201. doi: 10.1093/g3journal/jkag201 (PMC13643764; doi:10.1093/g3journal/jkag201)
Supplement: Supplementary file 1 [file jkag201_supplementary_data.zip › Supplementary_Figures_G3-2026-406973.pdf]

Supplementary Figures for

A Multiplex Droplet Digital PCR Assay for Chromosome Copy Number Determination in *Candida albicans*

Eli Isael Maciel, Sylvain Ursuegui, Sana Ahmed-Seghir, Corinne Maufrais, Stéphanie Roy, Cecile Gautier, Philipp Brandt, Cécile Jovelet, Laras Pitayu, Iuliana V. Ene

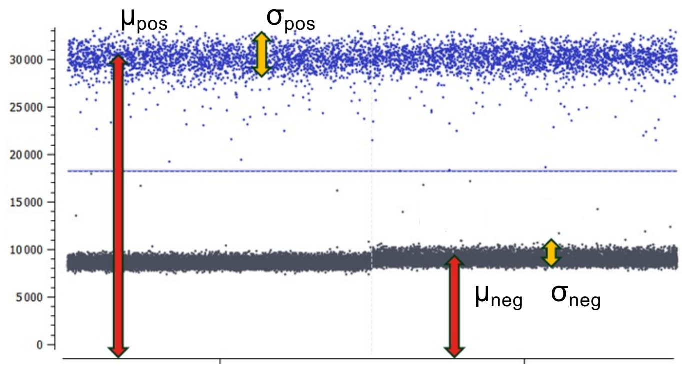

Separability score:

$$S = \frac{\mu_{pos} - \mu_{neg}}{\sqrt{\frac{p}{p-1}} * \sigma_{pos} + \sqrt{\frac{q}{q-1}} * \sigma_{neg}}$$

μ_{pos} = mean of the positive signals
μ_{neg} = mean of the negative signals
σ_{pos} = standard deviation of the positive signals
σ_{neg} = standard deviation of the negative signals
p = number of positive signals
q = number of negative signals

Supplementary Figure 1. Separability score used to quantify droplet-cluster resolution. Illustration of how the separability score (S) is calculated from the mean (μ) and standard deviation (σ) of positive and negative droplet fluorescence distributions, with the total numbers of positive (p) and negative (q) droplets indicated. Higher S values indicate better separation between positive and negative droplets and improved robustness of thresholding.

a

	euploid A		Chr1,4,5,7 3X	
	simplex	multiplex	simplex	multiplex
Chr1	2.35	3.05	0.99	1.16
Chr2	1.31	1.14	0.87	1.15
Chr3	0.79	0.92	0.79	0.97
Chr4	0.44	0.35	0.69	0.57
Chr5	1.25	1.26	1.10	1.10
Chr6	0.56	0.41	0.00	0.00
Chr7	0.88	1.04	0.96	0.97
ChrR	1.01	0.64	0.93	0.48

Chromosome arm R:L ratio

0 1 2 3

b

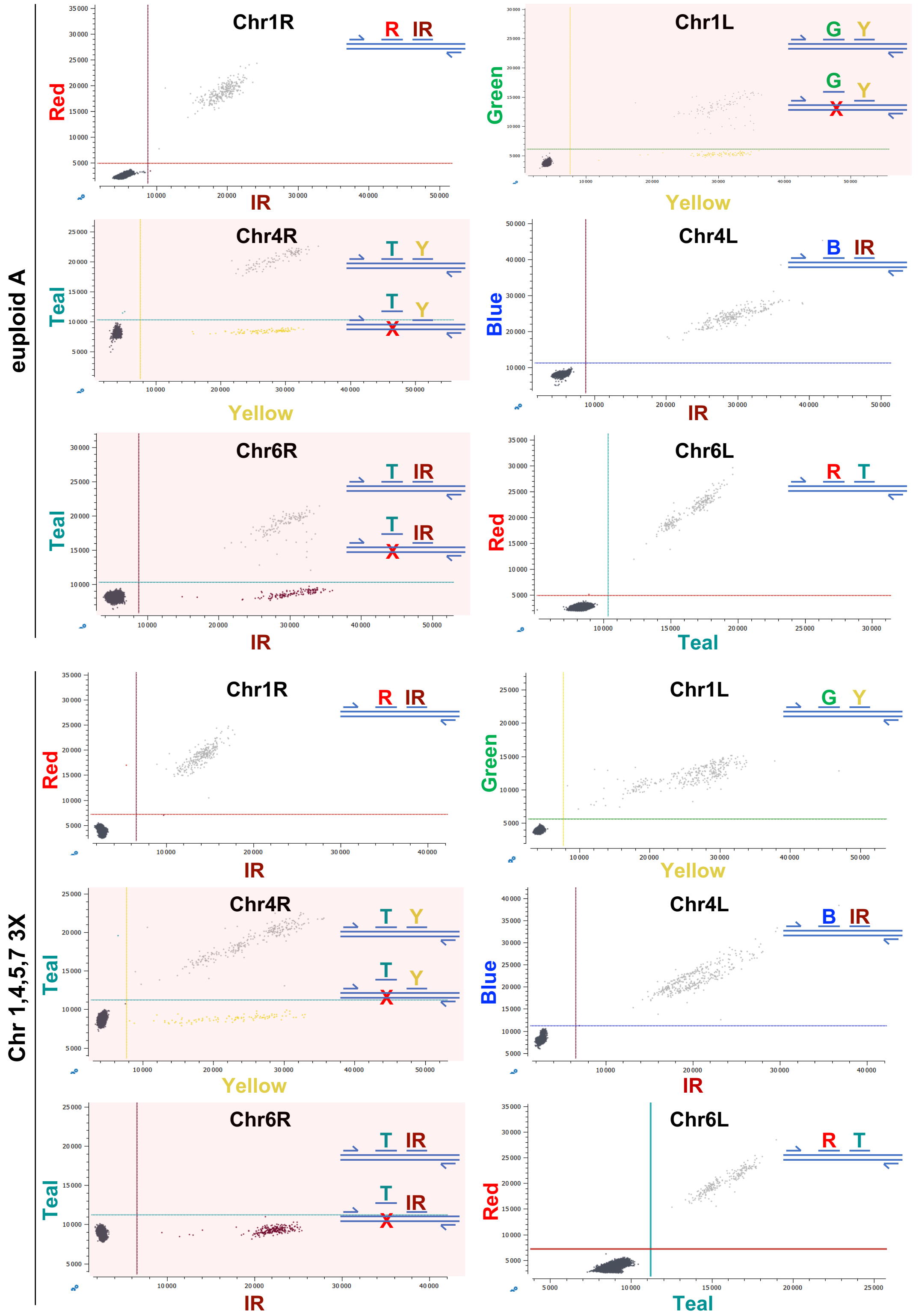

Supplementary Figure 2. Strain-dependent chromosome arm imbalances reveal probe-specific underperformance. (A) Heatmap of chromosome arm R:L ratios for isolates SC5314 (euploid A) and CAY8836-SC (Chr1,4,5,7, 3X) measured in simplex and multiplex. For each chromosome, the ratio was calculated as the copy-number estimate for the right-arm (R) target divided by the left-arm (L) target; values near 1 indicate balanced detection of both arms, whereas deviations reflect arm-specific bias (notably for Chr1, Chr4, Chr6, and ChrR). (B) Representative 2D amplitude (dot) plots from simplex reactions for arm targets showing the underlying probe behavior in euploid A (top) and Chr1,4,5,7, 3X (bottom) isolates. For Chr1L, Chr4R, and Chr6R, one probe in the original non-competing dual-probe pair yielded inefficient detection (shown by the crossed-out probe), reducing the expected double-positive population and inflating single-color droplets. In contrast, the corresponding partner-arm targets (Chr1R, Chr4L, Chr6L) show the expected well-separated positive clusters in their assigned channel pairs.

a

		Chromosome arm															
		1L	1R	2L	2R	3L	3R	4L	4R	5L	5R	6L	6R	7L	7R	RL	RR
10 min	euploid A	6.50	6.10	8.70	5.80	6.90	7.20	5.60	4.90	5.50	6.10	6.10	4.70	3.30	6.40	5.10	6.90
	ChrR 3X	6.20	5.40	8.80	6.90	8.10	7.50	5.90	5.30	4.70	6.40	5.90	4.50	3.30	6.30	5.30	7.30
	Chr1,6 3X	5.80	6.30	8.40	5.40	7.20	7.40	5.50	5.30	5.40	5.30	5.90	4.90	3.30	6.80	4.60	6.90
	Chr1,4,5,7 3X	5.30	5.70	9.30	6.20	8.00	8.10	5.80	5.10	5.50	5.50	5.90	4.60	3.20	5.70	4.90	6.90
	Chr6,R 4X	6.10	5.30	9.80	7.20	8.40	8.20	5.30	5.80	4.50	7.00	7.40	4.20	3.20	5.70	5.70	7.80
15 min	euploid A	9.30	5.60	11.40	10.00	9.30	9.90	6.40	8.00	6.40	11.10	7.60	5.00	3.10	8.40	7.40	8.60
	ChrR 3X	7.80	5.30	10.80	9.20	9.90	9.10	6.40	6.50	5.30	8.90	7.40	4.40	3.20	6.70	6.60	7.90
	Chr1,6 3X	7.70	5.50	9.30	7.00	8.80	8.10	5.60	5.90	5.60	6.70	7.20	4.90	3.20	7.40	5.20	6.80
	Chr1,4,5,7 3X	9.40	5.60	11.50	10.60	9.30	10.90	6.90	7.60	6.40	10.90	8.00	5.40	3.30	8.20	7.40	7.90
	Chr6,R 4X	8.40	4.70	11.00	9.20	9.70	9.00	5.70	8.00	5.30	9.40	7.70	4.30	3.20	6.60	6.80	7.70

Separability score

4 8 12

b

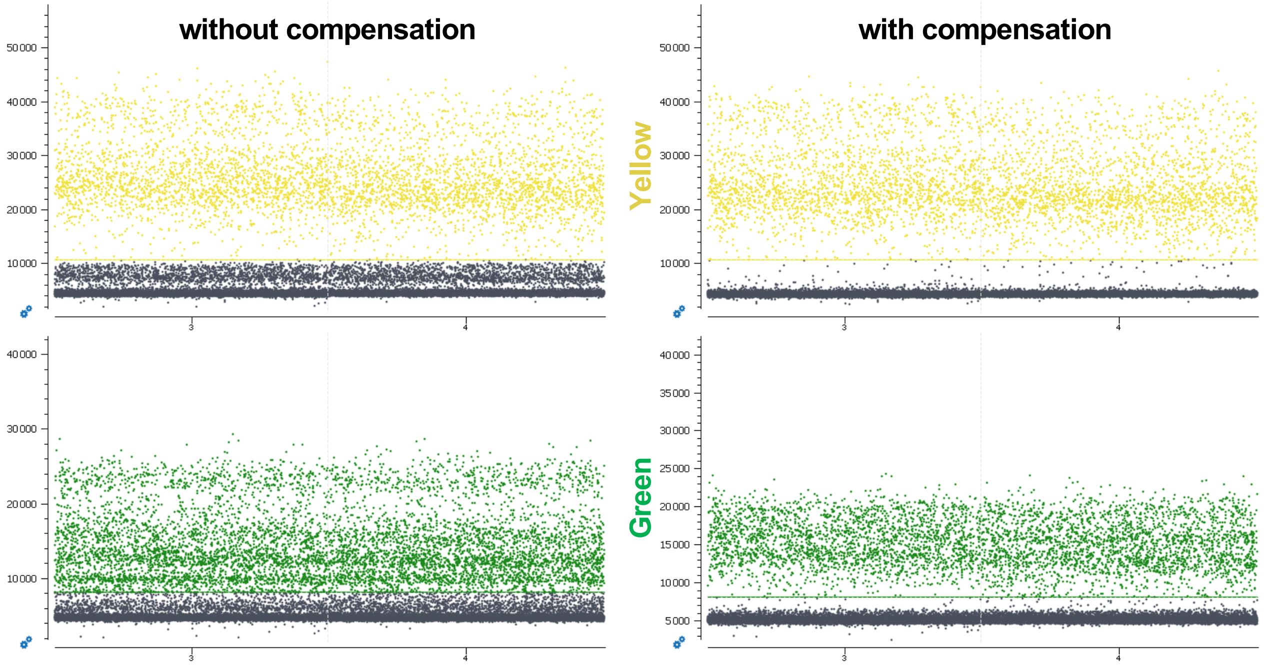

c

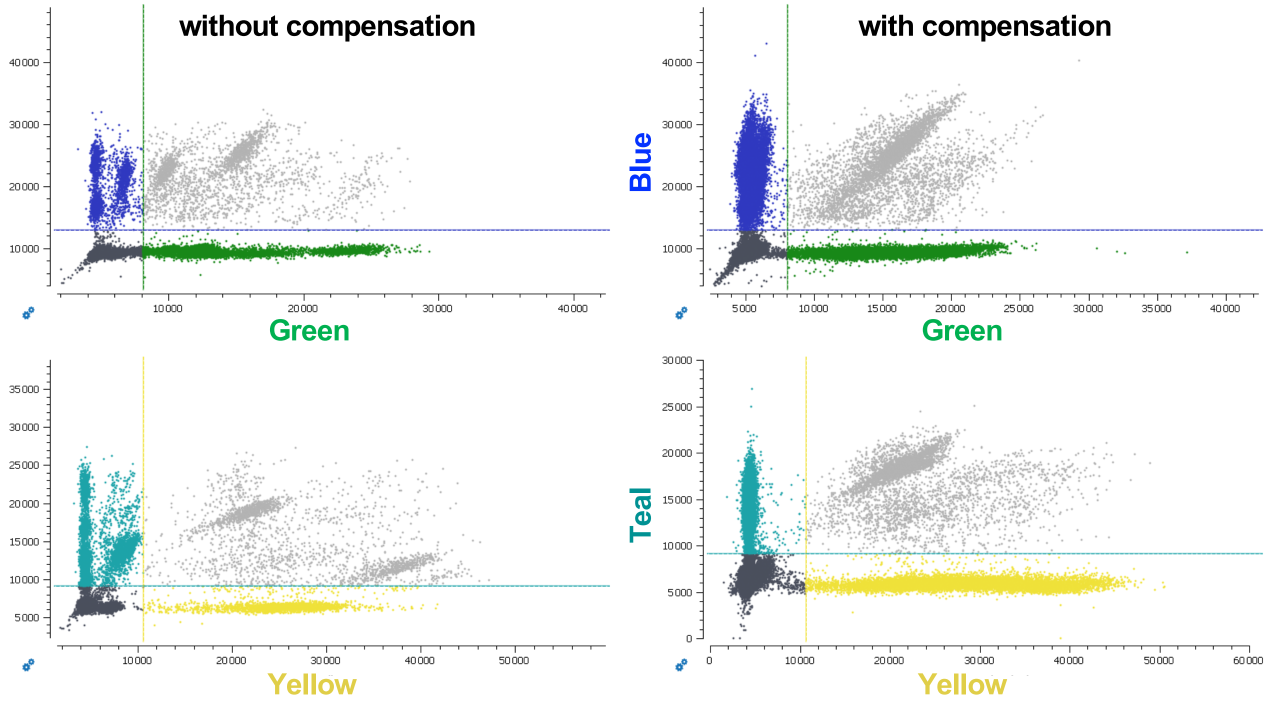

Supplementary Figure 3. Final elongation time and spillover compensation improve cluster separation in multiplex reactions. (A) Heatmap of separability scores for each chromosome-arm target (columns) across representative isolates, comparing a 10 min versus 15 min final elongation step. Higher scores indicate better separation between positive and negative droplet populations. (B) Representative 1D amplitude plots in the yellow and green channels before and after applying spillover compensation. Compensation yields a cleaner separation of positive (colored) and negative (gray) droplet populations. (C) Representative 2D amplitude (dot) plots (Blue vs Green; Teal vs Yellow) before and after compensation. Spillover correction restores the expected cluster geometry and increases separation between negative (gray) and positive (colored) droplet populations, supporting more reliable droplet calling.

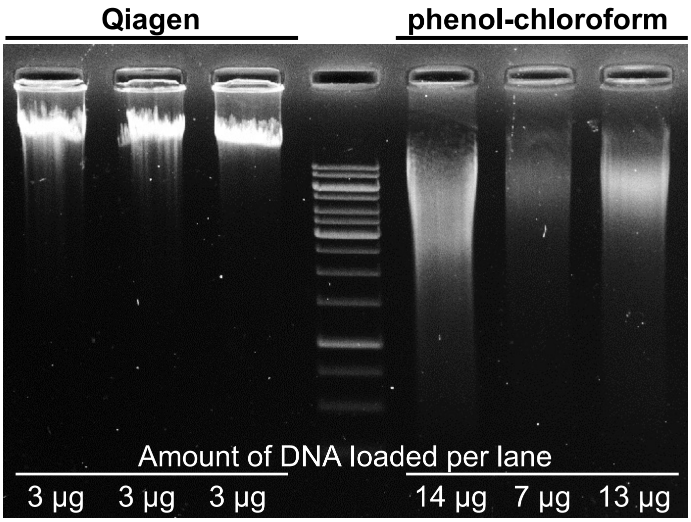

Supplementary Figure 4. Comparison of DNA integrity from phenol-chloroform and column-based extraction. Agarose-gel electrophoresis comparing genomic DNA extracted via Qiagen Genomic-tip (column-based) and phenol-chloroform methods. DNA isolated using phenol-chloroform appears more fragmented and less consistent in yield, supporting the use of column-purified DNA for reproducible ddPCR performance.

a

Chromosome arm

Extraction / input	Isolate	1L	1R	2L	2R	3L	3R	4L	4R	5L	5R	6L	6R	7L	7R	RL	RR
phenol chloroform 10 pg	euploid A	1.01	0.47	0.10	0.69	0.51	0.87	0.20	1.13	0.27	0.01	0.90	1.53	0.26	0.12	3.48	0.35
	Chr3 1X	0.47	0.07	1.50	0.16	1.10	1.81	0.16	0.56	1.90	2.51	0.56	0.95	1.41	1.10	1.59	1.88
	ChrR 3X	0.11	0.08	0.23	0.14	0.13	0.09	0.06	0.23	0.42	0.17	0.21	0.30	0.70	0.31	0.40	0.83
	Chr3,6 3X	0.03	0.36	1.16	0.53	1.27	0.35	0.70	1.41	0.17	0.12	0.79	0.61	0.70	0.34	2.26	1.09
	Chr6,R 4X	1.21	0.90	0.08	0.28	0.16	0.45	1.46	0.08	0.69	0.84	0.68	1.17	0.70	0.17	1.21	0.86
Qiagen 10 pg	euploid A	0.10	0.10	0.12	0.10	0.14	0.03	0.04	0.19	0.24	0.17	0.07	0.17	0.19	0.25	0.09	0.34
	Chr3 1X	0.39	0.20	0.13	0.32	0.52	0.14	0.20	0.36	1.15	0.21	0.18	0.87	0.23	0.26	0.11	0.58
	ChrR 3X	0.29	0.21	0.02	0.12	0.16	0.19	0.11	0.41	0.39	0.12	0.08	0.13	0.15	0.08	0.08	0.12
	Chr3,6 3X	0.09	0.07	0.08	0.05	0.21	0.08	0.32	0.09	0.06	0.13	0.12	0.12	0.04	0.03	0.16	0.16
	Chr6,R 4X	0.18	1.61	1.82	0.05	0.11	0.78	0.24	0.27	1.41	1.31	2.12	2.82	0.78	1.05	2.14	1.18
Qiagen 30 pg	euploid A	0.10	0.17	0.07	0.07	0.01	0.16	0.19	0.10	0.05	0.16	0.16	0.11	0.04	0.07	0.25	0.10
	Chr3 1X	0.13	0.17	0.15	0.06	0.19	0.13	0.06	0.18	0.19	0.24	0.09	0.12	0.10	0.16	0.24	0.08
	ChrR 3X	0.12	0.11	0.02	0.13	0.13	0.10	0.05	0.16	0.09	0.13	0.16	0.26	0.06	0.06	0.33	0.09
	Chr3,6 3X	0.18	0.37	0.11	0.02	0.18	0.31	0.02	0.04	0.13	0.09	0.22	0.24	0.24	0.12	0.50	0.05
	Chr6,R 4X	0.08	0.50	0.21	0.14	0.17	0.08	0.12	0.28	0.27	0.33	0.27	0.31	0.10	0.30	0.54	0.03

CNV standard deviation (color scale: 0, 1, 2)

b

Chromosome arm

Extraction / input	Isolate	1L	1R	2L	2R	3L	3R	4L	4R	5L	5R	6L	6R	7L	7R	RL	RR
phenol chloroform 10 pg	euploid A	0.10	0.08	0.12	0.11	0.14	0.09	0.14	0.10	0.11	0.11	0.14	0.13	0.08	0.05	0.09	0.09
	Chr3 1X	0.07	0.15	0.06	0.12	0.04	0.04	0.12	0.13	0.09	0.18	0.09	0.08	0.18	0.16	0.10	0.14
	ChrR 3X	0.46	0.55	0.64	0.55	0.48	0.44	0.61	0.57	0.92	0.56	0.59	0.55	0.41	0.23	0.66	0.85
	Chr3,6 3X	0.12	0.07	0.15	0.09	0.23	0.14	0.18	0.12	0.11	0.11	0.11	0.22	0.15	0.01	0.09	0.14
	Chr6,R 4X	0.02	0.10	0.09	0.13	0.04	0.05	0.07	0.13	0.10	0.16	0.12	0.20	0.12	0.04	0.20	0.16
Qiagen 10 pg	euploid A	1.05	1.20	1.22	1.27	1.22	1.25	1.23	1.17	1.11	1.19	1.29	1.37	1.21	1.29	1.20	1.25
	Chr3 1X	0.94	0.91	0.99	0.93	0.49	0.56	1.05	0.91	0.67	0.85	0.64	0.96	0.83	0.84	1.03	0.87
	ChrR 3X	1.08	1.11	1.04	1.16	1.04	1.05	1.04	1.07	1.02	1.19	0.99	1.10	1.17	1.13	1.66	1.57
	Chr3,6 3X	1.33	1.53	1.45	1.57	2.33	2.30	1.48	1.37	1.43	1.45	2.10	2.32	1.54	1.57	1.51	1.40
	Chr6,R 4X	1.64	1.89	1.60	1.71	1.75	1.68	1.72	1.47	1.61	1.41	1.49	2.25	1.45	1.57	2.46	2.21
Qiagen 30 pg	euploid A	2.17	2.21	2.34	2.32	2.17	2.17	2.36	2.24	2.29	2.30	2.31	2.47	2.31	2.43	2.44	2.25
	Chr3 1X	1.98	2.47	2.15	2.20	1.08	1.07	2.49	2.57	1.90	1.43	0.99	2.37	1.84	1.95	2.47	2.01
	ChrR 3X	2.12	2.86	2.19	2.46	1.98	2.25	2.37	2.03	2.28	2.01	1.94	2.66	2.11	2.04	4.01	2.98
	Chr3,6 3X	2.16	2.91	2.22	2.40	3.38	3.50	2.23	2.23	2.34	2.15	3.00	4.08	2.04	2.21	2.97	2.27
	Chr6,R 4X	2.45	3.75	2.65	2.87	2.32	2.60	2.84	2.79	2.73	2.05	1.94	4.14	2.18	2.13	4.76	3.18

Fraction of positive droplets (%) (color scale: 0, 1, 2, 3)

c

Chromosome arm

Extraction / input	Isolate	1L	1R	2L	2R	3L	3R	4L	4R	5L	5R	6L	6R	7L	7R	RL	RR
phenol chloroform 10 pg	euploid A	1.40	1.98	2.40	1.87	2.35	1.27	2.85	2.80	2.19	2.01	1.96	1.74	1.82	1.26	3.87	2.13
	Chr3 1X	1.67	3.06	1.95	2.11	1.23	1.73	2.11	2.62	2.68	1.77	1.38	0.67	3.00	2.77	2.90	1.33
	ChrR 3X	1.89	1.93	2.51	1.81	1.73	1.73	2.12	2.07	2.94	2.05	2.34	2.20	1.71	1.16	2.44	2.78
	Chr3,6 3X	1.98	0.94	3.22	1.02	2.90	2.44	2.50	3.00	1.92	1.88	1.24	3.16	2.89	0.44	3.00	1.42
	Chr6,R 4X	0.86	2.22	1.66	2.20	0.68	1.11	1.82	2.34	2.07	2.59	1.91	4.01	1.92	0.69	3.14	2.60
Qiagen 10 pg	euploid A	1.88	2.01	1.93	2.14	2.07	2.06	2.00	1.86	1.91	2.06	2.15	2.11	2.10	1.96	1.93	1.88
	Chr3 1X	2.26	1.85	2.15	2.28	1.34	1.30	2.22	1.80	2.13	2.02	1.80	2.61	2.20	1.69	2.28	2.26
	ChrR 3X	1.86	2.00	1.87	2.16	1.94	1.83	1.79	1.74	2.04	2.26	1.83	1.87	2.22	2.00	3.03	2.91
	Chr3,6 3X	1.78	1.92	1.97	2.13	3.07	3.02	1.99	1.80	1.84	1.87	2.87	3.04	2.02	2.04	1.93	1.77
	Chr6,R 4X	2.93	3.95	3.72	2.98	3.13	2.34	2.98	2.74	1.84	2.08	2.40	5.37	3.60	2.05	5.26	3.07
Qiagen 30 pg	euploid A	1.95	2.21	2.00	2.00	1.89	1.94	2.02	1.96	1.88	1.90	2.05	2.03	1.98	2.06	2.27	1.93
	Chr3 1X	1.97	2.06	2.01	2.10	1.13	0.99	2.38	2.36	1.73	2.01	1.83	2.12	2.24	1.90	2.12	1.94
	ChrR 3X	1.93	2.32	2.00	2.33	1.90	1.96	2.05	1.96	1.84	1.94	1.91	2.09	1.99	1.80	3.36	2.72
	Chr3,6 3X	1.83	2.00	1.93	2.13	2.91	2.75	1.94	1.97	1.84	1.97	2.81	3.05	1.95	1.92	2.27	1.96
	Chr6,R 4X	2.88	3.26	2.80	3.26	2.82	2.80	3.10	3.08	2.74	2.91	3.48	4.05	2.93	2.45	4.78	3.55

Chromosome copy number (color scale: 1, 2, 3)

Supplementary Figure 5. Effects of DNA extraction method and DNA input amount on the performance of the 16-plex assay across euploid and aneuploid isolates. (A) Standard deviation of chromosome copy-number estimates across biological replicates as a measure of technical variance. (B) Fraction of positive droplets per reaction/condition, reflecting effective amplifiable template availability. (C) Chromosome copy-number estimates under each condition, highlighting false-positive calls under suboptimal preparation.

a

Chromosome arm

	1L	1R	2L	2R	3L	3R	4L	4R	5L	5R	6L	6R	7L	7R	RL	RR
**none**	0.23	0.25	0.07	0.24	0.18	0.04	0.21	0.63	0.09	0.21	0.40	0.07	0.10	0.09	0.24	0.17
**EcoRI**	0.05	0.07	0.32	0.34	0.24	0.09	0.04	0.17	0.16	0.16	0.27	0.12	0.06	0.02	0.20	0.19
**BamHI**	0.12	0.23	0.09	0.09	0.24	0.07	0.32	0.11	0.13	0.35	0.16	0.28	0.11	0.10	0.15	0.21
**HindIII**	0.33	0.31	0.27	0.22	0.10	0.23	0.23	0.13	0.28	0.20	0.30	0.16	0.14	0.35	0.04	0.11
**PstI**	0.17	0.02	0.18	0.19	0.26	0.14	0.14	0.07	0.07	0.10	0.17	0.09	0.25	0.06	0.20	0.07
**PstI+BamHI**	0.07	0.24	0.10	0.11	0.28	0.07	0.08	0.27	0.15	0.29	0.15	0.14	0.16	0.33	0.16	0.26
**PstI+EcoRI**	0.14	0.03	0.18	0.06	0.15	0.01	0.20	0.16	0.15	0.07	0.07	0.11	0.08	0.18	0.19	0.17
**PstI+HindIII**	0.10	0.10	0.12	0.10	0.14	0.03	0.04	0.19	0.24	0.17	0.07	0.17	0.19	0.25	0.09	0.34
**BamHI+EcoRI**	0.18	0.10	0.11	0.12	0.23	0.07	0.12	0.10	0.18	0.11	0.07	0.19	0.20	0.11	0.09	0.18
**BamHI+HindIII**	0.14	0.11	0.42	0.42	0.03	0.12	0.15	0.08	0.07	0.16	0.13	0.07	0.28	0.11	0.36	0.19
**HindIII+EcoRI**	0.04	0.19	0.05	0.11	0.28	0.25	0.18	0.25	0.29	0.10	0.16	0.15	0.22	0.14	0.33	0.11

CNV standard deviation

0 1 2

b

Chromosome arm

	1L	1R	2L	2R	3L	3R	4L	4R	5L	5R	6L	6R	7L	7R	RL	RR
**none**	5.82	6.25	8.86	7.99	8.47	8.01	7.22	5.45	5.84	9.70	6.17	6.22	3.14	8.13	5.50	6.47
**EcoRI**	5.93	6.30	9.42	7.40	8.72	7.93	6.69	6.05	5.46	9.33	6.29	5.92	3.10	7.85	5.42	6.65
**BamHI**	7.16	8.69	10.89	8.49	9.96	8.78	8.06	6.79	6.98	10.24	7.93	6.97	3.35	8.04	6.80	7.26
**HindIII**	6.45	7.59	10.02	6.48	9.40	7.30	7.33	5.80	5.50	7.76	6.89	6.12	3.35	7.80	5.17	6.07
**PstI**	7.57	9.13	9.86	8.01	9.82	7.87	8.35	6.67	6.04	9.63	8.61	6.82	3.25	8.36	6.44	7.11
**PstI+BamHI**	6.58	7.95	10.85	7.59	9.70	7.82	7.71	6.80	5.83	9.91	6.93	6.22	3.26	8.38	5.86	7.39
**PstI+EcoRI**	6.84	7.71	9.50	8.88	9.50	8.49	7.56	6.12	6.14	10.96	7.53	6.14	3.26	9.06	6.46	7.90
**PstI+HindIII**	8.12	8.22	11.36	10.13	11.15	10.53	9.15	7.17	7.17	12.98	8.23	7.21	3.32	10.61	6.46	8.34
**BamHI+EcoRI**	5.28	6.81	9.37	7.02	8.53	7.34	6.88	5.29	5.19	8.41	6.37	5.95	3.44	7.47	4.81	6.12
**BamHI+HindIII**	5.95	7.52	8.77	6.10	8.06	8.00	6.68	5.28	5.49	7.08	6.34	5.89	3.17	7.17	4.31	6.32
**HindIII+EcoRI**	6.21	7.25	9.53	7.28	9.79	7.99	7.52	5.57	5.34	9.31	6.78	6.29	3.16	7.94	5.49	6.48

Separability score

4 8 12

c

Chromosome arm

	1L	1R	2L	2R	3L	3R	4L	4R	5L	5R	6L	6R	7L	7R	RL	RR
**none**	1.08	1.16	0.93	0.97	0.89	0.87	1.10	1.00	0.78	0.78	0.80	0.81	0.89	0.91	0.90	0.75
**EcoRI**	1.22	1.37	1.28	1.32	1.30	1.26	1.37	1.25	1.26	1.34	1.30	1.29	1.33	1.28	1.36	1.17
**BamHI**	0.83	0.84	0.69	0.76	0.87	0.84	0.98	0.86	0.88	0.87	0.83	0.86	0.94	0.85	0.87	0.85
**HindIII**	0.89	1.08	1.01	1.14	1.11	1.00	1.14	1.05	0.89	0.99	1.03	0.98	0.99	1.01	1.16	0.91
**PstI**	0.94	1.28	1.23	1.30	1.18	1.19	1.25	1.13	1.17	1.27	1.21	1.26	1.45	1.31	1.40	1.19
**PstI+BamHI**	0.87	0.92	0.97	0.99	0.93	0.90	1.02	0.96	0.91	0.89	0.88	0.99	0.91	0.97	1.02	0.91
**PstI+EcoRI**	1.22	1.30	1.27	1.14	1.19	1.17	1.24	1.16	1.19	1.26	1.35	1.25	1.27	1.21	1.26	1.10
**PstI+HindIII**	0.81	0.86	0.85	0.91	0.89	0.89	0.86	0.81	0.82	0.88	0.93	0.94	0.89	0.90	0.85	0.87
**BamHI+EcoRI**	1.14	1.12	1.20	1.24	1.14	1.16	1.31	1.12	1.14	1.15	1.19	1.27	1.19	1.23	1.17	1.17
**BamHI+HindIII**	1.02	1.20	1.25	1.23	1.18	1.12	1.21	1.15	1.12	1.20	1.17	1.21	1.16	1.14	1.31	1.00
**HindIII+EcoRI**	1.17	1.23	1.20	1.20	1.18	1.16	1.32	1.16	1.27	1.24	1.21	1.27	1.22	1.19	1.36	1.10

Fraction of positive droplets (%)

0 1 2 3

d

Chromosome arm

	1L	1R	2L	2R	3L	3R	4L	4R	5L	5R	6L	6R	7L	7R	RL	RR
**none**	2.39	2.55	2.07	2.21	2.03	1.95	2.43	2.09	1.76	1.80	1.90	1.83	1.97	2.00	2.03	1.72
**EcoRI**	1.91	2.11	2.17	2.15	2.09	1.97	2.11	1.92	1.96	2.13	1.97	1.97	2.06	1.98	2.09	1.75
**BamHI**	2.05	1.90	1.69	1.75	2.11	1.93	2.41	2.01	2.03	2.16	1.89	2.00	2.23	1.95	2.09	2.04
**HindIII**	1.62	2.09	2.19	2.25	2.15	1.92	2.30	2.05	1.66	2.00	1.94	1.86	1.91	1.95	2.25	1.73
**PstI**	1.92	2.04	2.16	2.13	2.04	1.82	2.06	1.78	1.92	2.10	1.86	2.00	2.45	2.07	2.22	1.91
**PstI+BamHI**	1.82	1.83	2.13	2.12	2.09	1.92	2.18	1.97	1.92	1.95	1.82	2.05	2.01	2.16	2.10	1.86
**PstI+EcoRI**	2.05	2.13	2.02	1.87	1.92	1.94	2.12	1.96	1.95	2.08	2.24	2.06	2.08	1.95	2.03	1.86
**PstI+HindIII**	1.88	2.01	2.13	2.14	2.07	2.06	2.00	1.86	1.91	2.06	2.15	2.11	2.10	1.96	1.93	1.88
**BamHI+EcoRI**	1.91	1.88	2.11	2.13	2.03	1.96	2.27	1.90	1.88	2.00	2.03	2.13	2.08	2.08	1.97	1.96
**BamHI+HindIII**	1.79	2.05	2.35	2.30	2.03	1.93	2.05	2.00	1.91	2.02	2.02	2.06	2.11	2.00	2.12	1.80
**HindIII+EcoRI**	1.95	2.03	1.94	2.01	2.05	1.84	2.21	1.92	2.21	2.09	2.07	2.06	2.11	1.94	2.17	1.80

Chromosome copy number

1 2 3

Supplementary Figure 6. Restriction-enzyme digestion improves droplet resolution and assay consistency. Performance metrics for undigested DNA versus digestion with single enzymes or enzyme combinations. Heatmaps summarize changes following SC5314 (euploid A) DNA digestion in (A) variability of chromosome copy-number estimates (SD across replicates), (B) droplet separability scores, (C) fraction of positive droplets, and (D) chromosome copy-number profiles.
